# Screen habits and effects on sensory profiles in 6- to 36-month-old toddlers

**DOI:** 10.1038/s41390-025-04024-x

**Published:** 2025-04-02

**Authors:** Estelle Gillioz, Edouard Gentaz, Fleur Lejeune

**Affiliations:** 1 Department of Psychology, University of Geneva, Geneva, Switzerland; 2 Swiss Center for Affective Sciences, University of Geneva, Geneva, Switzerland; 3 CNRS, Paris, France

## Abstract

**Background:**

The pervasive presence of screens in toddlers’ environments leads to earlier, longer, and more varied exposure to digital devices. Although they provide toddlers with developmentally inappropriate visual and auditory stimulations, only one study has investigated the effect of these screens on their sensory development. The current research, therefore, explores the links between screen use habits and sensory profiles in 6- to 36-month-old toddlers.

**Methods:**

Data were collected online using two questionnaires: a questionnaire designed to gather information regarding the use of screens within households and the Dunn’s Infant/Toddler Sensory Profile 2 Questionnaire to assess the toddlers’ sensory profile.

**Results:**

Significant differences in sensory processing based on screen exposure were found: 6- to 18-month-old toddlers showed higher sensitivity and registration scores related to greater direct screen exposure, while 19- to 36-month-old toddlers principally showed higher seeking scores related to greater direct and background screen exposure.

**Conclusion:**

These behavioral patterns suggest that excessive screen exposure may impact sensory processing, reducing opportunities for active, multisensory interactions essential for development, emphasizing the need for guidelines to manage screen use in early childhood to promote optimal sensory and cognitive development.

**Impact:**

- Early screen exposure and sensory processing of 6- to 36-month-old toddlers are linked
- Potential risks of excessive screen exposure time in toddlerhood could include behavioral problems and hyperactivity through sensory over-responsivity patterns
- Monitoring and managing screen use in early childhood is crucial for optimal development and to reduce the risk of behavioral disorders in a digital age

## Introduction

The rapid evolution of digital technologies has significantly transformed the environments in which children grow up today. Households are now multi-equipped, possessing an average of no fewer than seven screens (for example, in France^1^ and in Switzerland^2^). Consequently, children are exposed from a very young age to various types of screens, including televisions, tablets, smartphones, and computers, and it has been repeatedly shown that their daily screen exposure time continues to increase.^3,4^ On average, toddlers between 0 and 3 years are exposed to screens for thirty minutes to three hours almost every day, and this even increased following the COVID-19 pandemic.^5–7^ This time spent in front of screens is time lost to other activities and reduces the time and opportunities available for the interpersonal experiences necessary for children’s socio-emotional and cognitive development, as well as the time they could devote to exploring their environment with their senses.^8,9^ A large majority of parents in Switzerland also say they spend time in front of screens while their infant is present in the room.^2^ Toddlers are therefore subjected to background screen exposure while their parents watch television or use screens in front of them, which can lead to disruptions in interactions (the phenomenon termed technoference^10^). As a result, children’s learning opportunities and interactions are reduced. Empirical evidence suggests that screen exposure has an impact on various components of psychological development,^11–18^ and that the phenomenon of technoference can have an impact on the attachment bond and reduce the quality and quantity of parent-child interactions.^19,20^ Yet these interactions are essential for toddlers to develop appropriate social, emotional, behavioral, and language skills.^21^ We have also recently shown that toddlers under the age of three with greater screen exposure time demonstrated weaker tactile exploration skills, but sustained attention and prosocial behaviors of these toddlers were significantly better in those who experienced interactive co-viewing during exposure, underling once again the importance of parent-child interactions.^22,23^

Furthermore, screens provide intense auditory and visual stimulations, but often in a passive manner: a child is usually seated motionless behind a lit screen, with which no interaction is possible. This contrasts with the active and multisensory interactions crucial for healthy sensory development, which is an essential component of overall toddlers’ development, influencing their abilities to interact with their environment and acquire cognitive and sensori-motor skills.^24,25^ Sensory experiences serve as the primary sources of information for young children. Depending on the sensory stimuli perceived and identified in their environment, such as sounds, textures, colors, and movements, their reactions and behaviors will vary. For instance, tactile and bodily sensations aid children in exploring their surroundings and manipulating objects.^26^ These object interactions facilitate increasingly complex, flexible, and controlled motor actions^27^ and enhance their physical and symbolic capacities.^28,29^ However, to achieve this, children must be able to manage the tactile sensory stimulations that arise from such exploration. The ability to regulate responses to sensory stimuli perceived in the environment, regardless of their nature, is crucial for avoiding sensory overload or, conversely, understimulation. This regulation also allows children to focus and calm themselves by filtering and prioritizing sensory stimuli.^30^ Conversely, children who face sensory challenges in their daily lives, such as sensory over-responsivity, may encounter difficulties in their daily activities, particularly in fully participating in activities that would promote their development.^31,32^

As evidenced by various research findings, the first three years of toddlers’ lives are crucial for the development of the different cognitive and sensori-motor skills associated with the development and refining of sensory system and processing abilities. In fact, it has been shown that children with Autism Spectrum Disorders,^33^ Attention deficit/hyperactivity disorder,^34,35^ Down syndrome^36,37^ or other developmental disorders^38–40^ generally present different sensory processing difficulties.

It is, and will be, therefore essential to identify any potential sensory processing dysfunctions as early as possible, as well as factors that may be linked to these deficits. It is effectively well established that early childhood experiences are essential determinants of health, well-being, and the development of various cognitive and social skills later in life.^41^ As a result, the increasing use of screens by young children has raised concerns among researchers and healthcare professionals. Studies have shown that excessive screen exposure can be associated with a range of developmental issues (for example of review^13,16,42^) and that they provide developmentally inappropriate visual and auditory stimulations.^43,44^ In this context, identifying the link between screen use and potential sensory processing dysfunctions in very young children could provide insights for professionals aiming to support optimal child development in an increasingly digital environment. To date, only one study has examined the link between toddlers’ sensory profiles and their direct screen exposure time.^45^ This study was a prospective one, using data from the National Children’s Study (*N* = 1471). The authors examined the link between early screen exposure (measured at 12, 18, and 24 months of age) and sensory processing outcomes among toddlers at approximatively 33 months of age. Using the different quadrant scores from the Dunn’s Infant/Toddler Sensory Profile, the results showed that screen time at 12, 18, and 24 months of age was associated with atypical sensory processing outcomes at 33 months of age. For example, greater screen exposure time at 24 months of age was associated with an increased risk of high sensation seeking later in development. Nevertheless, only the direct screen exposure time of toddlers is reported in this study, using a single question asked to caregivers. The toddlers’ background screen exposure time was, therefore, not considered, nor were the different sensory and behavioral section scores measured by the Infant/Toddler Sensory Profile.

The current study aims to explore the effects of direct, background, and total screen exposure time on the sensory profiles of 6- to 36-month-old toddlers, as assessed by Dunn’s Infant/Toddler Sensory Profile 2 Questionnaire.^46,47^ Since data are cross-sectional, toddlers were separated into different groups to shed light on the potential impact of screen exposure on sensory profile trajectories. Based on these elements, we put forward two main hypotheses. Firstly, in line with the results found in the Heffler et al. study,^45^ we hypothesize that toddlers with a greater use of screens will show higher scores on each of the four quadrants scores compared to those with lower exposure. Furthermore, we hypothesize that toddlers with a greater use of screens will show significantly different scores of the sensory and behavioral sections scores from those of toddlers with a lower use of screens.

## Methods

### Participants

We collected data from 159 toddlers in this study: forty-six between 6 and 18 months (*M* = 11 months, *SD* = 3), thirty-eight between 19 and 24 months (*M* = 20 months, *SD* = 3), and seventy-five between 25 and 36 months (*M* = 30 months, *SD* = 4). Families were recruited through the child’s daycare facility, and there were no exclusion criteria concerning toddlers. However, parents had to speak French to answer the questionnaires. The families’ socio-economic level is calculated from the age, the level of education, and the professional category of both parents. The resulting socio-economic position index^48^ divides families into five social classes: lower (1–35; 20.2% of the general population in Switzerland), middle-lower (36–54; 20%), middle (55–67; 19.6%), upper-middle (68–80; 20.9%) and upper (>80; 19.3%). According to this index, the average families’ socio-economic level in our sample is at the entrance to the Swiss upper-middle class (*M* = 70.4, *SD* = 12.5).

### Procedure

This study was part of a larger project investigating links between screen habits and several aspects of child development approved by the Ethics Committee of the Faculty of Psychology and Educational Sciences at the University of Geneva.

Data collection using questionnaires took place in the first part of the study. After receiving a signed informed consent form from the participating parents, they were asked to complete the questionnaire assessing screen habits and The Infant/Toddler Sensory Profile 2 online (Qualtrics, Provo, UT). The questionnaires were offered in French only. The total completion time for both questionnaires was around thirty minutes.

### Questionnaires

#### Screen habits

Toddlers’ screen habits were measured using a questionnaire created specifically for this purpose. It was designed to gather information regarding the use of screens within households, particularly by toddlers. It comprises various questions divided into four main sections. In the first section, we collect general information about the toddlers and their families, such as their age, the parents’ professional activities, the household composition, etc. The second section primarily focuses on the number of screens in the household and the parents’ daily use of these different screens. The third section is the most substantial part of the questionnaire. It covers all aspects of toddlers’ screen use and viewing habits. For example, we ask parents to report days, times, and types of screen exposure (i.e., direct use and background exposure) for their toddler, as well as their involvement during viewing. The final section asks parents about their general knowledge regarding the potential effects of screens on children’s development, as well as the current recommendations made and disseminated to parents.

Three screen exposure outcomes were computed from parent responses for analysis: (1) The direct screen exposure time of toddlers in minutes per day; (2) The background screen exposure time in minutes per day, which corresponds to the number of minutes per day the parent or someone else spent on screens when the toddler was present in the room; (3) The total screen exposure time, giving by the addition of these two first variables.

#### Infant/Toddler Sensory Profile 2

This questionnaire is a judgement-based caregiver questionnaire that provides a standard method for measuring toddlers’ behavioral responses to various sensory stimuli. It consists of fifty-four items describing age-appropriate behaviors or responses to various sensory experiences within the different sensory systems, such as “My child startles more easily than others of the same age (e.g., barking dogs, children yelling)” or “My child avoids touching rough, cold, or sticky surfaces (e.g., rugs, countertops)”.^46^ For each statement, the principle caregiver of the toddler is asked to indicate on a five-point Likert-type scale the frequency with which their toddler exhibits these behaviors. This scale ranges from 5 (almost always/90% or more of the time) to 1 (almost never/10% or less of the time) and offer the possibility to put 0 (does not apply) if the caregivers are unable to answer because they have not observed such behaviors or believe that it does not apply to their toddler.

Using the number of points obtained for each answer, items are grouped into seven sensory sections: general information processing (items 1–10, range from 0 to 50 points), auditory processing (items 11–17, 0–35), visual processing (items 18–23, 0–30), tactile processing (items 26–31, 0–30), vestibular/movement processing (items 36–40, 0–25), oral sensory processing (items 42–48, 0–35), and behavioral responses related to sensory information processing (items 49–54, 0–30). Based on the scores obtained, it is possible to determine if the child exhibits sensory vulnerabilities, either generally or within these specific domains. Additionally, depending on the toddlers’ response to different items throughout the questionnaire, it is possible to calculate the toddlers’ varying patterns across the four quadrants of sensory responsiveness featured in the model: seeking (0–35), avoiding (0–55), sensitivity (0–65) and registration (0–55). A high score on each of these components does not mean that the sensory reactions and associated behaviors are better or more adapted, but it indicates a greater occurrence of the corresponding behaviors. For instance, a child scoring higher than peers on the sensitivity component will be more reactive to various sensory inputs from their environment, which could distract them and interfere with their participation in daily activities (Table 1). These scores describe the toddlers’ general behavioral tendencies to sensory stimuli, while sensory section scores (such as auditory or visual processing scores) measure the specific responses to particular types of stimuli.

**Table 1:** Meaning and examples of possible behaviors when the score is high in the four quadrants of sensory responsiveness.

	Meaning	Example of a typical toddler profile
**Scores**		
Seeking	Active search for sensory stimuli.	Constantly seek interaction and sensory stimulation. Described as more restless, noisy, and prone to boredom.
Avoiding	Avoidance or rejection of sensory stimuli.	Try to reduce or avoid sensory experiences perceived as unpleasant or overwhelming. Described as a solitary child who likes to be left alone and prefers quiet environments.
Sensitivity	Overreaction or heightened sensitivity to sensory stimuli.	Quickly alerted by changes in the environment and perceive certain sensations overwhelming. Sensitive to details and could appear easily distracted.
Registration	Lack of response to sensory stimuli.	Do not readily perceive sensory stimuli in their environment. Described as uninterested in their environment, disengaged, and apathetic.

### Data analysis

To establish the independent variables related to screen habits, we divided toddlers in each age group into two categories. For direct screen exposure time, we adhered to international guidelines (i.e., no screens before age one-and-a-half^49^), similar to other studies.^50,51^ On this basis, toddlers aged 6–18 months were classified into two groups: those who had not yet been exposed to screens (group 0, GR0) and those who had already been exposed (group 1, GR1). For the toddlers aged between 19 and 36 months, two groups were created before separating them again into subgroups according to screen habits: one group made up of toddlers aged between 19 and 24 months, and a second one made up of toddlers aged between 25 and 36 months. Since the first three years of life are marked by significant developmental changes, separating these toddlers into these two distinct groups enables us to analyze screen-related developmental issues in greater detail. For both age groups, international guidelines (i.e., less than one hour per day^49^) are respected in the majority of cases. We therefore based our analysis on actual screen time, and separated the toddlers into two groups according to their average direct screen exposure time: those with direct screen exposure time above the average were placed in group 1 (GR1), while those with exposure below or equal to the average were placed in group 0 (GR0). For background screen exposure time and total screen exposure time, no specific guidelines were given. Therefore, toddlers in each age group were divided into two groups based on their average daily screen time: those with above-average screen exposure were assigned to group 1 (GR1), and those with below or equal to average exposure were assigned to group 0 (GR0). All these subgroup splits have been based on the average, as some toddlers in our sample are highly exposed to screens, and these values are important to consider since data collection by questionnaire generally leads to an underestimation of time spent in front of screens, and the average of the general population is higher than that of our sample.

We conducted data analysis using IBM SPSS Statistics, Version 29.0.2.0. We first ran Kolmogorov–Smirnov to assess the normality of the data. All data were normally distributed, with the exception of seeking scores and avoiding scores for children between 25 and 36 months. We therefore ran Independent-samples *t*-tests whenever possible or Mann–Whitney tests when the data failed to meet assumptions of parametric analysis, using screen habits (direct screen exposure time, background screen exposure time, and total screen exposure time) as independent variables and the different quadrant and section scores as dependent variables. In addition, to assess the evolution of screen habits between age groups, we ran the Kruskal–Wallis test and post-hoc comparisons when results were significant using Dunn’s method with a Bonferroni correction. A significance level of 0.05 was used for all statistical tests.

## Results

### Effect of screen habits on sensory profile scores for each age group

Regarding the effects of direct, background and total screen exposure time on sensory profile scores, significant differences are observed for some of quadrants and sections scores, depending on toddlers’ age.

#### 6- to 18-month-old toddlers

The quadrant score and the section scores depending on screen habits for each subgroup of 6- to 18-month-old toddlers are presented in Tables 2–4.

**Table 2:** Means and standard deviations of quadrant and section scores depending on direct screen exposure time for each subgroup (GR0 vs. GR1) of 6- to 18-month-old toddlers.

	Direct screen exposure time			
	GR0 (*N* = 22)	GR1 (*N* = 24)	*p*	Cohen’s d
**Screen time (min/day)**	0 (0)	13 (15)	—	—
**Quadrants**				
Seeking	26 (6)	26 (7)	0.981	0.007
Avoiding	9 (6)	11 (6)	0.227	−0.361
Sensitivity	17 (6)	22 (8)	0.027	−0.675
Registration	8 (6)	12 (6)	0.070	−0.547
**Sensory and behavioral sections**				
General	11 (5)	13 (5)	0.267	−0.332
Auditory	6 (4)	6 (5)	0.959	−0.015
Visual	16 (5)	17 (8)	0.625	−0.145
Touch	5 (4)	7 (4)	0.100	−0.496
Movement	14 (4)	17 (5)	0.016	−0.737
Oral	8 (3)	12 (6)	0.009	−0.790
Behavioral	7 (5)	8 (3)	0.511	−0.195

The groups were formed on the basis of the international guidelines: group 1 (GR1) includes toddlers who had already been exposed to screens, and group 0 (GR0) includes toddlers who had not yet been exposed to screens.

**Table 3:** Means and standard deviations of quadrant and section scores depending on background screen exposure time for each subgroup (GR0 vs. GR1) of 6- to 18-month-old toddlers.

	Background screen exposure time			
	GR0 (*N* = 36)	GR1 (*N* = 10)	*p*	Cohen’s d
**Screen time (min/day)**	56 (13)	174 (101)	—	—
**Quadrants**				
Seeking	25 (7)	26 (5)	0.747	−0.116
Avoiding	10 (6)	12 (7)	0.445	−0.275
Sensitivity	19 (7)	22 (9)	0.197	−0.468
Registration	9 (6)	13 (6)	0.129	−0.553
**Sensory and behavioral sections**				
General	12 (5)	12 (6)	0.961	−0.018
Auditory	6 (5)	7 (3)	0.820	−0.082
Visual	15 (6)	21 (6)	0.025	−0.830
Touch	6 (4)	8 (5)	0.212	−0.453
Movement	15 (5)	15 (4)	0.965	0.016
Oral	15 (5)	13 (9)	0.269	−0.622
Behavioral	7 (4)	11 (6)	0.008	−0.999

Groups were formed on the basis of average background screen exposure time (*M* = 82, *SD* = 68): group 1 (GR1) includes toddlers with above-average background screen exposure time, and group 0 (GR0) includes toddlers with background screen exposure time below or equal to the average.

**Table 4:** Means and standard deviations of quadrant and section scores depending on total screen exposure time for each subgroup (GR0 vs. GR1) of 6- to 18-month-old toddlers.

	Total screen exposure time			
	GR0 (*N* = 34)	GR1 (*N* = 12)	*p*	Cohen’s d
**Screen time (min/day)**	59 (13)	169 (110)	—	—
**Quadrants**				
Seeking	25 (7)	27 (5)	0.470	−0.244
Avoiding	10 (6)	12 (7)	0.214	−0.424
Sensitivity	18 (7)	23 (8)	0.039	−0.607
Registration	9 (6)	14 (6)	0.019	−0.817
**Sensory and behavioral sections**				
General	12 (5)	13 (6)	0.530	−0.213
Auditory	6 (5)	7 (3)	0.431	−0.267
Visual	15 (6)	20 (6)	0.015	−0.852
Touch	6 (3)	9 (5)	0.042	−0.702
Movement	15 (5)	16 (4)	0.531	−0.212
Oral	10 (4)	13 (8)	0.195	−0.616
Behavioral	7 (4)	10 (5)	0.016	−0.844

Groups were formed on the basis of average total screen exposure time (*M* = 88, *SD* = 74): group 1 (GR1) includes toddlers with above-average total screen exposure time, and group 0 (GR0) includes toddlers with total screen exposure time below or equal to the average.

The results indicated significant differences in sensitivity score according to direct screen exposure time, *t*(44) = −2.285, *p* = 0.027 and total screen exposure time, *t*(44) = −1.807, *p* = 0.039. The results also indicated a significant difference in registration score depending on the total screen exposure time, *t*(44) = −2.432, *p* = 0.019. Moreover, the results indicated significant differences in movement and oral components according to direct screen exposure time, respectively, *t*(44) = −2.498, *p* = 0.016 and, *t*(44) = −2.743, *p* = 0.009. They also indicated significant differences in visual and behavioral components according to background screen exposure time, respectively, *t*(44) = −2.322, *p* = 0.025 and, *t*(44) = −2.795, *p* = 0.008. Finally, looking at total screen exposure time, the results indicated significant differences in visual, *t*(44) = −2.537, *p* = 0.015, behavioral, *t*(44) = −2.515, *p* = 0.016, and touch components, *t*(44) = −2.092, *p* = 0.042.

#### 19- to 24-month-old toddlers

The quadrant score and the section scores depending on screen habits for each subgroup of 19- to 24-month-old toddlers are presented in Tables 5–7.

**Table 5:** Means and standard deviations of quadrant and section scores depending on direct screen exposure time for each subgroup (GR0 vs. GR1) of 19- to 24-month-old toddlers.

	Direct screen exposure time			
	GR0 (*N* = 29)	GR1 (*N* = 9)	*p*	Cohen’s d
**Screen time (min/day)**	7 (8)	95 (46)	—	—
**Quadrants**				
Seeking	25 (6)	30 (3)	0.021	−0.919
Avoiding	13 (5)	15 (5)	0.321	−0.384
Sensitivity	20 (8)	22 (8)	0.576	−0.215
Registration	11 (6)	12 (5)	0.775	−0.110
**Sensory and behavioral sections**				
General	14 (5)	17 (6)	0.217	−0.479
Auditory	8 (5)	9 (4)	0.680	−0.158
Visual	12 (6)	17 (6)	0.026	−0.887
Touch	7 (4)	7 (2)	0.720	−0.138
Movement	17 (3)	18 (3)	0.734	−0.131
Oral	8 (5)	9 (4)	0.956	−0.021
Behavioral	9 (4)	10 (4)	0.553	−0.229

Groups were formed on the basis of average direct screen exposure time (*M* = 28, *SD* = 44): group 1 (GR1) includes toddlers with above-average direct screen exposure time, and group 0 (GR0) includes toddlers with direct screen exposure time below or equal to the average.

**Table 6:** Means and standard deviations of quadrant and section scores depending on background screen exposure time for each subgroup (GR0 vs. GR1) of 19- to 24-month-old toddlers.

	Background screen exposure time			
	GR0 (*N* = 26)	GR1 (*N* = 12)	*p*	Cohen’s d
**Screen time (min/day)**	60 (17)	228 (97)	—	—
**Quadrants**				
Seeking	25 (6)	27 (6)	0.457	−0.263
Avoiding	14 (4)	12 (6)	0.245	0.413
Sensitivity	21 (8)	19 (9)	0.364	0.321
Registration	13 (6)	9 (5)	0.058	0.684
**Sensory and behavioral sections**				
General	15 (5)	14 (6)	0.744	0.115
Auditory	9 (4)	7 (5)	0.208	0.447
Visual	13 (5)	13 (7)	0.890	−0.049
Touch	7 (3)	5 (3)	0.051	0.705
Movement	18 (3)	17 (3)	0.231	0.425
Oral	9 (6)	7 (3)	0.387	0.305
Behavioral	9 (4)	10 (4)	0.694	−0.138

Groups were formed on the basis of average background screen exposure time (*M* = 113, *SD* = 96): group 1 (GR1) includes toddlers with above-average background screen exposure time, and group 0 (GR0) includes toddlers with background screen exposure time below or equal to the average.

**Table 7:** Means and standard deviations of quadrant and section scores depending on total screen exposure time for each subgroup (GR0 vs. GR1) of 19- to 24-month-old toddlers.

	Total screen exposure time			
	GR0 (*N* = 26)	GR1 (*N* = 12)	*p*	Cohen’s d
**Screen time (min/day)**	73 (30)	289 (120)	—	—
**Quadrants**				
Seeking	25 (5)	28 (6)	0.211	−0.445
Avoiding	14 (4)	13 (6)	0.596	0.186
Sensitivity	21 (8)	20 (9)	0.861	0.062
Registration	12 (6)	11 (5)	0.646	0.162
**Sensory and behavioral sections**				
General	15 (5)	14 (6)	0.744	0.115
Auditory	9 (5)	8 (5)	0.786	0.096
Visual	12 (6)	15 (7)	0.246	−0.411
Touch	7 (4)	6 (3)	0.663	0.153
Movement	18 (3)	17 (4)	0.505	0.235
Oral	8 (6)	9 (4)	0.875	−0.055
Behavioral	9 (4)	11 (4)	0.303	−0.364

Groups were formed on the basis of average total screen exposure time (*M* = 141, *SD* = 124): group 1 (GR1) includes toddlers with above-average total screen exposure time, and group 0 (GR0) includes toddlers with total screen exposure time below or equal to the average.

The results indicated a significant difference in seeking score according to direct screen exposure time, *t*(36) = −2.408, *p* = 0.021. Moreover, the results indicated a significant difference in visual component score depending on the direct screen exposure time, *t*(36) = −2.325, *p* = 0.026.

#### 25- to 36-month-old toddlers

The quadrant score and the section scores depending on screen habits for each subgroup of 25- to 36-month-old toddlers are presented in Tables 8–10.

**Table 8:** Means and standard deviations of quadrant and section scores depending on direct screen exposure time for each subgroup (GR0 vs. GR1) of 25- to 36-month-old toddlers.

	Direct screen exposure time			
	GR0 (*N* = 57)	GR1 (*N* = 18)	*p*	Cohen’s d
**Screen time (min/day)**	14 (10)	116 (101)	—	—
**Quadrants**				
Seeking	24 (7)	25 (7)	0.484	−0.190
Avoiding	14 (6)	14 (6)	0.813	−0.064
Sensitivity	17 (7)	16 (8)	0.533	0.169
Registration	10 (5)	13 (8)	0.072	−0.494
**Sensory and behavioral sections**				
General	15 (5)	16 (5)	0.217	−0.479
Auditory	8 (5)	9 (5)	0.486	−0.189
Visual	12 (6)	14 (7)	0.335	−0.262
Touch	6 (4)	7 (4)	0.318	−0.272
Movement	15 (5)	15 (4)	0.814	0.064
Oral	6 (5)	7 (4)	0.413	−0.223
Behavioral	8 (4)	9 (4)	0.389	−0.234

Groups were formed on the basis of average direct screen exposure time (*M* = 39, *SD* = 66): group 1 (GR1) includes toddlers with above-average direct screen exposure time, and group 0 (GR0) includes toddlers with direct screen exposure time below or equal to the average.

**Table 9:** Means and standard deviations of quadrant and section scores depending on background screen exposure time for each subgroup (GR0 vs. GR1) of 25- to 36-month-old toddlers.

	Background screen exposure time			
	GR0 (*N* = 53)	GR1 (*N* = 22)	*p*	Cohen’s d
**Screen time (min/day)**	58 (26)	181 (71)	—	—
**Quadrants**				
Seeking	23 (7)	27 (6)	0.043	−0.493
Avoiding	14 (6)	14 (5)	0.747	−0.082
Sensitivity	17 (8)	18 (7)	0.703	−0.097
Registration	10 (6)	13 (7)	0.060	−0.484
**Sensory and behavioral sections**				
General	15 (6)	16 (4)	0.744	0.115
Auditory	8 (5)	9 (4)	0.385	−0.222
Visual	12 (6)	15 (6)	0.145	−0.373
Touch	6 (4)	6 (4)	0.671	−0.108
Movement	15 (5)	16 (3)	0.338	−0.245
Oral	6 (5)	7 (4)	0.548	−0.153
Behavioral	7 (4)	10 (5)	0.014	−0.639

Groups were formed on the basis of average background screen exposure time (*M* = 94, *SD* = 71): group 1 (GR1) includes toddlers with above-average background screen exposure time, and group 0 (GR0) includes toddlers with background screen exposure time below or equal to the average.

**Table 10:** Means and standard deviations of quadrant and section scores depending on total screen exposure time for each subgroup (GR0 vs. GR1) of 25- to 36-month-old toddlers.

	Total screen exposure time			
	GR0 (*N* = 52)	GR1 (*N* = 23)	*p*	Cohen’s d
**Screen time (min/day)**	74 (29)	265 (120)	—	—
**Quadrants**				
Seeking	24 (7)	26 (7)	0.265	−0.281
Avoiding	14 (6)	14 (6)	0.901	−0.031
Sensitivity	17 (8)	17 (7)	0.885	−0.036
Registration	10 (6)	13 (7)	0.074	−0.455
**Sensory and behavioral sections**				
General	15 (6)	15 (4)	0.744	0.115
Auditory	8 (5)	8 (4)	0.584	−0.138
Visual	12 (6)	14 (6)	0.263	−0.283
Touch	6 (4)	6 (4)	0.540	−0.154
Movement	15 (5)	15 (4)	0.814	−0.059
Oral	6 (5)	7 (4)	0.350	−0.236
Behavioral	7 (4)	9 (5)	0.061	−0.476

Groups were formed on the basis of average total screen exposure time (*M* = 133, *SD* = 113): group 1 (GR1) includes toddlers with above-average total screen exposure time, and group 0 (GR0) includes toddlers with total screen exposure time below or equal to the average.

The results indicated a significant difference in seeking score according to background screen exposure time, *U* = 756.5, *p* = 0.043. Moreover, the results indicated a significant difference in behavioral component score depending on the background screen exposure time, *t*(73) = −2.521, *p* = 0.014.

### Additional results

Table 11 presented the evolution of screen habits as a function of the toddlers’ age. The results indicated that there was a significant difference in direct screen exposure time across age, *χ*^2^ (2, *N* = 159) = 32.883, *p* = <0.001. Post-hoc comparisons indicated that the direct screen exposure time of toddlers between 6 and 18 months was significantly lower than that of toddlers between 19 and 24 months, *p* = 0.002, and toddlers between 25 and 36 months, *p* = <0.001, and that the direct screen exposure time of toddlers between 19 and 24 months was significantly lower than that of toddlers between 25 and 36 months, *p* = 0.043. The test also indicated that there was a significant difference in total screen exposure time across age, *χ*^2^ (2, *N* = 159) = 11.367, *p* = 0.003. Post-hoc comparisons indicated that the total screen exposure time of toddlers between 6 and 18 months was significantly lower than that of toddlers between 19 and 24 months, *p* = 0.011, and toddlers between 25 and 36 months, *p* = 0.001. However, there was no significant difference between the total screen exposure time of toddlers between 19 and 24 months and toddlers between 25 and 36 months, *p* = 0.807. Conversely, there were no significant differences between age groups in terms of background exposure time, *χ*^2^ (2, *N* = 159) = 3.567, *p* = 0.168.

**Table 11:** Evolution of screen habits between age groups.

Measures	6–18 months	19–24 months	25–36 months	*p*
*Screen habits*				
Direct screen exposure time (min/day)	7 (13)	28 (44)	39 (66)	<0.001
Background exposure time (min/day)	82 (68)	113 (96)	94 (71)	0.168
Total screen exposure time (min/day)	88 (74)	141 (124)	133 (113)	0.003

## Discussion

In the present study, we examined the links between the 6- to 36-month-old toddlers’ screen use habits, particularly their screen exposure time, and their sensory profile as assessed by Dunn’s Infant/Toddler Sensory Profile 2 Questionnaire.^46^ Our main hypotheses were (1) that toddlers with greater use of screen will have higher scores on each of the four quadrants scores compared to those with lower exposure, and (2) that these toddlers will also have significant differences in the different scores of the sensory and behavioral sections scores. First, our results showed that toddlers’ exposure increases with age, in line with the existing literature (for example, ref. ^2^), while their parents’ use of screens in their presence seems to be stable over the years, at over an hour and a half a day. Then, and most importantly, our results partially confirmed our hypotheses: significant differences were observed in some quadrants and sections scores as a function of direct screen exposure time, background screen exposure time, and total screen exposure time, depending on the toddlers’ age.

### 6- to 18-month-old toddlers

Toddlers between 6 and 18 months appeared to be the most affected by early screen use on their sensory processing: those who had already been directly exposed to screens and for a longer period of time reacted more quickly and strongly to different sensory stimuli.

The results first indicated higher scores in the sensitivity component in toddlers who had already been directly and intentionally exposed to screens and whose total screen exposure time was greater, although their screen exposure time was very low compared to screen time reported in other studies.^52,53^ The sensitivity component refers to the child’s reactivity to various sensory stimuli in their environment. A higher sensitivity score indicates that these toddlers react more quickly and strongly to sensory stimuli. They are quickly alerted by changes in their environment and may perceive certain sensations as overwhelming. It is possible that the rapidity of images in children’s programs and the array of sounds and colors projected by screens cause sensory overload in these toddlers, subsequently increasing sensory over-reactivity behaviors. Additionally, the results indicated that it was primarily the movement and the oral components that were related to direct screen exposure. These are two components closely linked to environmental exploration. Children who score higher on the movement component are generally described as avoiding activities involving rapid movements or position changes. These children tend to move less, seek to avoid sensory stimulations, and consequently engage less in exploratory behaviors. Furthermore, children who score higher in the oral component are described as having difficulty bringing non-food objects to their mouths. However, putting an object in the mouth is the first tactile exploration strategy used by the infant in the first year of life.^54^ Early screen exposure could, therefore, impact the development of tactile exploration through sensory processes, adding that a child in front of screens is losing time that could be spent exploring their environment and engaging in various activities that promote sensory development and understanding of the world.^9^

Moreover, the results indicated a higher score in the registration component in toddlers between 6 and 18 months who had a greater total screen exposure time. Despite their quick reactions to different environmental sensory stimuli, toddlers with high registration scores exhibit a high detection threshold. Consequently, these toddlers do not readily perceive sensory stimuli in their surroundings. As a result, they are often characterized as uninterested in their environment and apathetic. It has been shown that children exhibiting such patterns have a decreased preference for all social activities,^55^ whereas those who are sensorily engaged from birth through parent-child interactions express a marked preference for tactile exploration and social stimuli, as well as social engagement with peers and strangers.^56^ It is thus possible that these toddlers, left alone in front of screens or regularly exposed to parental technoference and the interactional disruptions resulting from their parents’ screen use in their presence, experience fewer social interactions and are consequently less sensorily engaged through interactions with their environment, all the more so as the results showed that it was primarily the visual, touch, and behavioral components that were most affected. Since interactions and tactile exploration are fundamental to child development,^23,27,29,57^ this pattern of sensitivity could then affect various aspects of their development. Furthermore, some results of research argue that the age of first exposure to screens may be an important factor affecting toddlers’ development and cognition.^58^ This could also explain why toddlers who are exposed so early showed different sensory patterns, even if their direct screen exposure times are still limited.

### 19- to 36-month-old toddlers

Concerning the older toddlers, higher scores were observed for the seeking component according to direct and background screen exposure time.

A higher seeking score indicates that these toddlers interact more with their environment, actively search for sensory experiences, and engage in a multitude of exploratory behaviors. Consequently, these toddlers are often described as more turbulent, noisy, and easily bored. It may be due that these toddlers have been accustomed to high levels of sensory stimulation from a very young age, primarily due to their screen exposure: screens provide intense auditory and visual stimulations. Compared with screens, the physical environment may appear dull to the child who does not explore it. Additionally, parents often report using screens as babysitters in the majority of cases.^59^ This use does not leave room for boredom in the child’s development, as they are constantly stimulated by the colors and sounds emitted by screens.^60^ It is possible that children become accustomed to this vast array of stimuli from an early age and thus are constantly seeking stimulations, finding the real world less engaging.^61^

Toddlers with more seeking behaviors are also described as having greater difficulties predicting danger: they are more likely to run around, climb, and jump everywhere. These behaviors can put them in danger but also serve to attract their caregivers’ attention. Studies on the phenomenon of technoference show that parents’ use of screens in the presence of their child can lead them to engage in more dangerous behaviors to regain their attention.^10,62^ This could explain why toddlers between 19 and 36 months, who very often experience these interactional disconnects, are the ones who tend to engage in these seeking behaviors, while these are behaviors that cannot yet be seen in younger toddlers, who have not developed the necessary motor skills.^63,64^ Furthermore, the results concerning the sensory and behavioral section scores revealed that it was primarily the visual and the behavioral components that differed among the most exposed children in our sample compared to those who were less exposed. These findings thereby lend further support to these explanatory hypotheses since it is mainly visual search and behavior that are involved in the search for stimulation and attention from the parent.

Finally, all these results are also intriguing in light of scientific data linking screen exposure to behavioral problems and hyperactivity. It has been repeatedly shown that excessive direct screen exposure is associated with the development of externalizing disorders years later.^42,65,66^ Furthermore, children with attention-deficit/hyperactivity disorder often exhibit sensory over-responsivity and seek sensory input more actively,^34^ similar patterns to those observed in our sample of toddlers who were more frequently exposed to screens. Although it’s not possible to diagnose such a potential disorder at this young age, these sensory sensitivity patterns could, thus, be an early indicator related to early screen exposure. Similar findings in the prospective study by Heffler et al.^45^ on a larger sample of toddlers actually support these hypotheses: their results also suggested that early screen exposure could be a potential risk factor for the development of atypical sensory profiles (higher screen exposure time at 24 months of age was for example associated with increased risk of high sensation seeking later in development), which could have an impact on toddlers’ broader psychological development.

### Limitations and perspective

There are several limitations to the current study that could be considered in future studies. First, all data were collected by questionnaire. Although our questionnaire addresses the question of screen habits in different ways and considers the background screen exposure time of toddlers (a major limitation of the study by Heffler et al.^45^), parental self-reports might not be the best way to collect data, especially concerning screen habits. For reasons of social desirability, parents may, for example, underestimate the total number of minutes they spend in front of screens, as well as the number of occurrences of interactional disconnections caused by screens.^67,68^ Moreover, it has been shown that patterns of sensory over-reactivity become more evident when children enter school.^69^ This new environment demands a great deal of adaptation, as the social and physical novelty are often more stimulating than at home. Although the early years are crucial in a child’s development, and it is already possible to observe some significant differences according to early screen exposure, it could be interesting to analyze in a future study the sensory reactions of these children once they arrived at school.

## Conclusion

In all instances, the Infant/Toddler Sensory Profile 2 provides a way to capture the toddlers’ responses to sensory experiences and stimulations on a daily basis. When combined with other information about the toddler in context, typically their screen use habits, this information can be used to plan effective intervention promoting healthy developmental outcomes in the increasingly digital environment in which children grow up today. For example, these findings underline the importance of considering the impact of early screen exposure on the sensory processing and overall development of young children. Excessive screen exposure time not only reduces opportunities for active, multisensory interactions crucial for sensory development but also poses potential risks for developing behavioral problems and hyperactivity. As sensory sensitivity and seeking patterns can be early indicators of potential developmental issues, it is essential to monitor and manage screen use in early childhood to promote optimal sensory and cognitive development. In light of the growing presence of digital technologies in toddlers’ lives, this study provides valuable information for healthcare professionals, educators, and parents. It emphasizes the need for guidelines and interventions aimed at minimizing direct and background screen exposure time during the critical early years of development.

## Data Availability

The datasets generated during and/or analyzed during the current study are available from the corresponding author upon reasonable request.
